# Enhancement of the catalytic activity of Isopentenyl diphosphate isomerase (IDI) from *Saccharomyces cerevisiae* through random and site-directed mutagenesis

**DOI:** 10.1186/s12934-018-0913-z

**Published:** 2018-04-30

**Authors:** Hailin Chen, Meijie Li, Changqing Liu, Haibo Zhang, Mo Xian, Huizhou Liu

**Affiliations:** 1 CAS Key Laboratory of Bio-based Materials, Qingdao Institute of Bioenergy and Bioprocess Technology, Chinese Academy of Sciences, No. 189 Songling Road, Qingdao, 266101 People’s Republic of China; 2 Sino-Danish College, University of Chinese Academy of Sciences, No. 19(A) Yuquan Road, Beijing, 100049 People’s Republic of China

**Keywords:** Isopentenyl diphosphate isomerase, Random mutagenesis, Enzyme activity, Lycopene production, Mevalonate pathway

## Abstract

**Background:**

Lycopene is a terpenoid pigment that has diverse applications in the food and medicine industries. A prospective approach for lycopene production is by metabolic engineering in microbial hosts, such as *Escherichia coli*. Isopentenyl diphosphate isomerase (IDI, E.C. 5.3.3.2) is one of the rate-limiting enzymes in the lycopene biosynthetic pathway and one major target during metabolic engineering. The properties of IDIs differ depending on the sources, but under physiological conditions, IDIs are limited by low enzyme activity, short half-life and weak substrate affinity. Therefore, it is important to prepare an excellent IDI by protein engineering.

**Results:**

Directed evolution strategy (error-prone PCR) was utilized to optimize the activity of *Saccharomyces cerevisiae* IDI. Using three rounds of error-prone PCR; screening the development of a lycopene-dependent color reaction; and combinatorial site-specific saturation mutagenesis, three activity-enhancing mutations were identified: L141H, Y195F, and W256C. L141H, located near the active pocket inside the tertiary structure of IDI, formed a hydrogen bond with nearby β-phosphates of isopentenylpyrophosphate (IPP). Phe-195 and Cys-256 were nonpolar amino acids and located near the hydrophobic group of IPP, enlarging the hydrophobic scope, and the active pocket indirectly. Purified IDI was characterized and the result showed that the *K*_m_ of mutant IDI decreased by 10% compared with *K*_m_ of the parent IDI, and *K*_cat_ was 28% fold improved compared to that of the original IDI. Results of a fermentation experiment revealed that mutant IDI had a 1.8-fold increased lycopene production and a 2.1-fold increased yield capacity compared to wild-type IDI.

**Conclusion:**

We prepared an engineered variant of IDI with improved catalytic activity by combining random and site directed mutagenesis. The best mutants produced by this approach enhanced catalytic activity while also displaying improved stability in pH, enhanced thermostability and longer half-life. Importantly, the mutant IDI could play an important role in fed-batch fermentation, being an effective and attractive biocatalyst for the production of biochemicals.

**Electronic supplementary material:**

The online version of this article (10.1186/s12934-018-0913-z) contains supplementary material, which is available to authorized users.

## Background

**Fig. 1:**
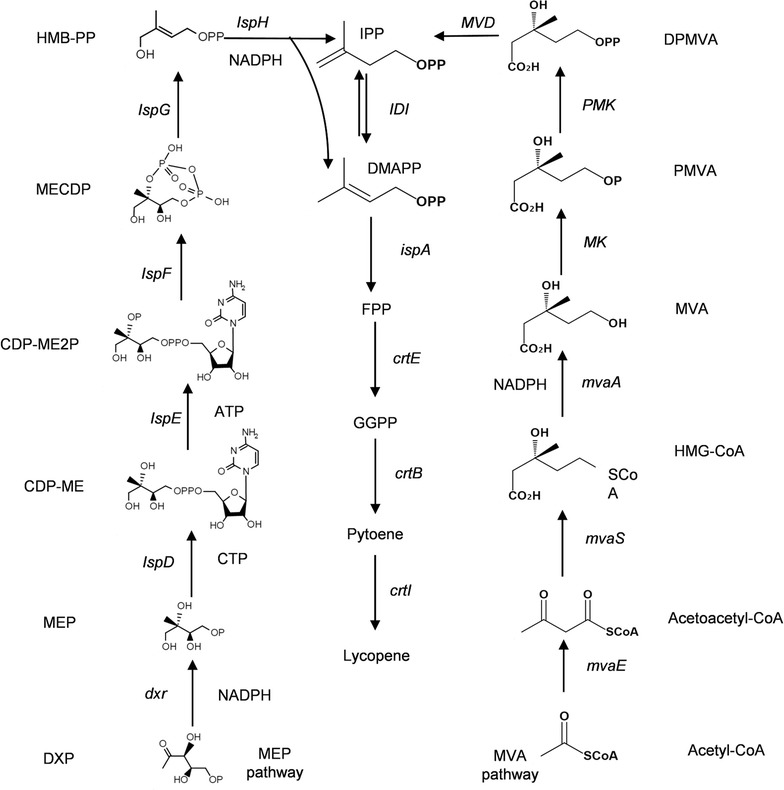
The MEP and MVA pathways. In the MVA pathway, a series of enzymes catalyze two molecules of acetyl-CoA into IPP, which is then isomerized by IDI to its isomer—DMAPP. In the MEP pathway, glyceraldehyde 3-phosphate and pyruvate are converted to IPP and DMAPP. At the same time, IDI as an isomerase, functions to balance the amount of IPP and DMAPP. Under the catalysis by the downstream enzymes, DMAPP enters the synthesis pathway of the target product

Lycopene is a widely used carotenoid in the healthcare product market due to its potent antioxidant properties and its links to reduced risk of prostate cancer in humans [1]. Given the recent development of metabolic engineering technologies, one promising approach is the introduction of constituent enzymes of the lycopene biosynthetic pathway into a heterologous host. Cellular lycopene can be synthesized by two different pathways, the mevalonate (MVA) pathway [2] and the 2-methyl-d-erythritol 4-phosphoric acid (MEP) pathway [3] (Fig. 1). The former is the main synthetic pathway and exists in the cytoplasm, while the latter exists in the chloroplast [4, 5]. Isopentenyl diphosphate isomerase (IDI, EC 5.3.3.2), which catalyzes the isomerization of isopentenylpyrophosphate (IPP) to dimethylallyl pyrophosphate (DMAPP), is a key rate-limiting enzyme for terpenoid biosynthesis [6]. Depending on different pathways, IDI plays a different but important role in the terpenoid biosynthesis pathway [7]. As reported, the overexpression *IDI* gene from *Escherichia coli* and *Bacillus licheniformis* in *E. coli* could greatly improve lycopene production [8, 9].

The properties of IDIs differ depending on the sources, but under physiological conditions, IDIs are limited by low enzyme activity, short half-life and weak substrate affinity [10]. Therefore, it is important to prepare an excellent IDI by protein engineering. We chose *Saccharomyces cerevisiae* IDI (CP008183) as the template, and successfully overexpressed it in *E. coli* [11, 12]. In the MVA pathway, 3-hydroxy-3-methyl glutaryl coenzyme A (HMG-CoA) was a key intermediate [13], and the accumulation of HMG-CoA would cause the toxicity to the bottom MVA pathway [14]. In this work, we integrated the bottom portion of the MVA pathway and the lycopene synthesis pathway by constructing a recombinant *E. coli* strain with the plasmids pET-CHL (containing the bottom MVA pathway) and pAC-LYC (containing key lycopene synthesis genes). An improved high-throughput IDI screening method was established, which not only removed the toxicity of HMG-CoA, but also used the lycopene-dependent color development reaction for selection [15]. Site-directed saturation mutagenesis was conducted to determine the optimal amino acid substitutions at the identified positions [16]. Combinations of site-specific mutations were investigated to identify which combination or combinations of mutations could further enhance IDI enzymatic activity.

## Results

### Screening for recombinant strains with high lycopene production

**Fig. 2:**
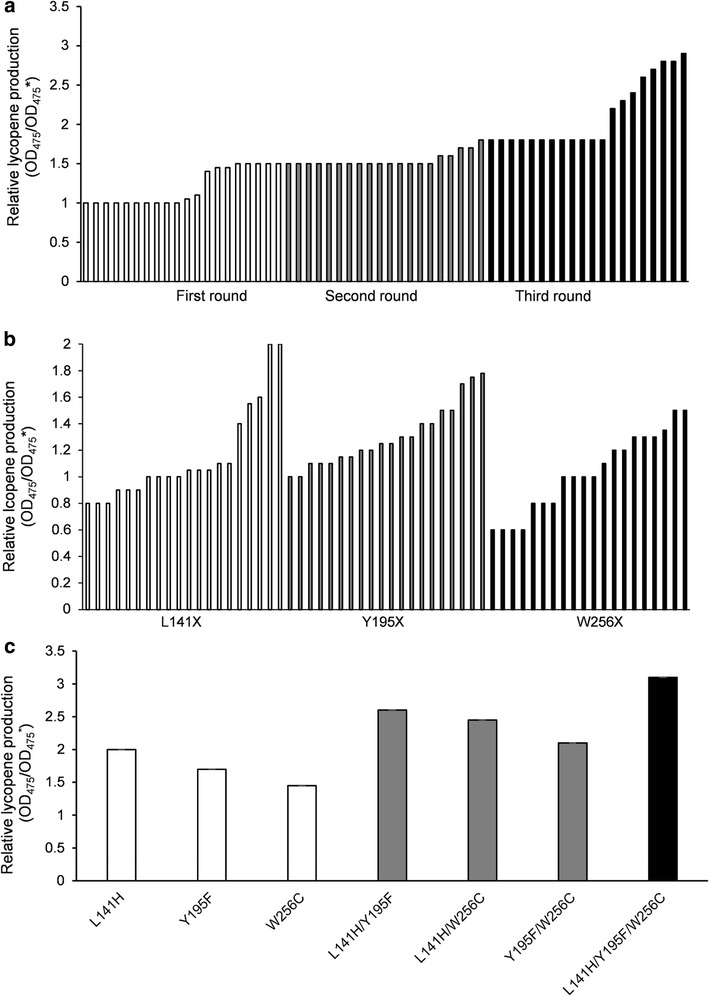
Screening results of three cycles of random mutagenesis (**a**); saturation mutagenesis at residues L141, Y195, and W256 (**b**); and critical amino acid substitutions by site-directed mutagenesis (**c**). **a** Twenty representative strains are shown for each sequential mutagenesis step. The average relative lycopene production of the mutants increased progressively with every round of mutagenesis. Strains with the highest relative lycopene production in each round were chosen for further analysis. **b** Twenty representative strains are shown for every position to identify the most advantageous amino acid substitution at these residues. These same colour columns represent different residual activities at the same position. **c** Single, double and triple mutation enzymes were constructed to determine which were sufficient and necessary for enhanced IDI activity. Every mutant was necessary for IDI and worked well with no interference. Wild-type IDI was used as the control (OD_475_^*^)

We performed three cycles of error-prone PCR, and the capacity of the mutation library was always 10,000–15,000 strains. Using the lycopene color development reaction as the primary screening method, 20 objective bacteria strains were obtained in each cycle (Fig. 2a).

Eight objective bacteria strains were identified in the first cycle of screening (Fig. 2a), the genomes of which were sequenced and used as a template to perform the second cycle of error-prone PCR. Five objective bacteria strains were identified in the second cycle (Fig. 2a), the genomes of which were sequenced and used as a template to carry out the third cycle of error-prone PCR. Eight objective bacteria strains emerged from the third cycle (Fig. 2a). These 21 strains were sequenced, 17 of which were determined to contain base mutations, and 14 strains contain amino acid mutations. The changes of bases had led to changes in the protein sequence (Additional file 1: Table S2).

### Site-directed saturation mutagenesis of residues L141, Y195, and W256

Substitutions L141, Y195 and W256 were identified as mutations capable of enhancing lycopene production. Separate saturation mutagenesis libraries were constructed using NNK codon degeneracy (20 amino acids/32 codons), which can encode all possible amino acids at positions L141, Y195 and W256 in IDI. Approximately 300 colonies from each of the three libraries were screened to ensure that all possible substitutions were assessed. It has been suggested that screening 94 NNK codon degeneracy mutagenesis colonies will yield a 95% probability of evaluating all possible 32 outcomes [17].

Five colonies from the V13 library displayed enhanced lycopene production (Fig. 2b), two of which contained histidine substitutions (L141H), while the remaining three contained lysine (L141K) and arginine (L141R), which were the substitutions identified in the consensus library. Amino acid analysis showed that alkaline amino acids were predominate in this screening assay. The L141H variant displayed 1.05-fold improvement in lycopene production compared to the wild type, while L141K and L141R showed 0.41-fold and 0.63-fold enhancement. 18 colonies from the Y195 library displayed enhanced lycopene production (Fig. 2b), and all contained substitutions of residues hydrophobic side chains: alanine, leucine, valine, isoleucine, and phenylalanine. Substitutions of Y195-to-Val, -Ile, -Leu or -Ala enhanced lycopene production between 0.22- and 0.49-fold compare to the wild type, although lycopene production was lower than that of Phe148 (which displayed a 0.71-fold increase) as determined in the validation assay. From the W256 screening library, four colonies, including W256C, displayed enhanced lycopene production (Fig. 2b). During the validation screen, variants with improved activity, W256F, W256L and W256I, were found to have a 0.11- to 0.29-fold increase in lycopene production in comparison with the wild type enzyme. However, W256C showed the highest increase in lycopene production (0.48-fold).

### Identification of critical IDI mutations

The optimal amino acid substitutions for IDI from the saturation mutagenesis screen were L141H, Y195F and W256C. Next, a combinatorial mutagenesis approach was used to identify the IDI variants with the largest increase in lycopene production.

L141H, Y195F and W256C showed 1.10-, 0.71- and 0.47-fold higher lycopene production compared with the wild type, respectively (Fig. 2c), indicating that these individual mutations were all beneficial and improved lycopene production. Moreover, L141H/Y195F, L141H/W256C and Y195F/W256C showed 2.03-, 1.65- and 1.14-fold higher lycopene production, respectively (Fig. 2c), indicating that double mutation combinations could further improve lycopene production. Furthermore, no double mutation combination decreased production. Finally, the triple mutant L141H/Y195F/W256C showed the highest improvement (3.13-fold of wild type) (Fig. 2c). In summary, the enhanced catalytic activity of mutant IDI resulted from additive mutations at residues 141, 195 and 256.

### Determination of kinetic parameters

The kinetic parameters of IDI and triple-mutant IDI (L141H/Y195F/W256C) were summarized in Table 1. The *K*_m_ of mutant IDI decreased by 10% from 41.5 ± 0.39 to 37.6 ± 0.17 μM [18]. Such a decrease indicated that the affinity of triple mutant IDI for substrate had increased, and the specific activity of the enzyme had risen. The *K*_cat_ value increased from 8.27 ± 0.06 to 10.57 ± 0.13 s^−1^. The catalytic efficiency of the enzyme was therefore enhanced from 0.20 to 0.28 s^−1^ μM^−1^.

**Table 1:** Kinetic parameters of IDI and IDI (L141H/Y195F/W256C)

Enzyme	*K*_m_ (μM)	*K*_cat_ (s^−1^)	*K*_cat_/*K*_m_ (s^−1^ μM^−1^)	Maximum enzyme activity (U/mg)
IDI	41.5 ± 0.39	8.27 ± 0.06	0.20	63.87 ± 1.09
IDI (L141H/Y195F/W256C)	37.6 ± 0.17	10.57 ± 0.13	0.28	161.60 ± 2.74

Triple-mutant IDI (L141H/Y195F/W256C) resulted in 1.40-fold improvement in the *K*_cat_/*K*_m_ value. Specific activity of IDI (L141H/Y195F/W256C) was 2.53-fold higher than that of IDI (Table 1). In addition, the expression levels of the wild type and mutated IDI were verified in our study. From Additional file 1: Figure S2, the wild type and mutated IDI had same expression levels. It proved that three mutations didn’t affect expression level of IDI, and the expression levels of IDI and IDI (L141H/Y195F/W256C) didn’t affect lycopene production.

### Enzymatic properties of wild-type and triple-mutant IDI

**Fig. 3:**
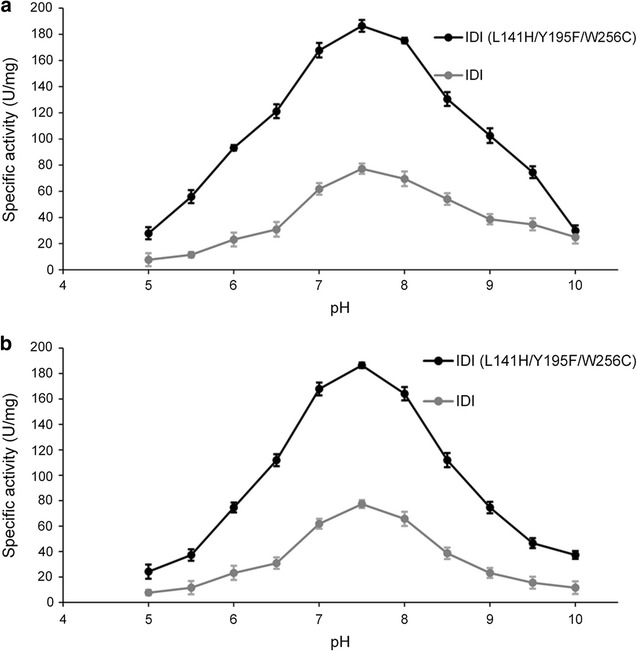
Effect of pH on the activity (**a**) and stability (**b**) of IDI (L141H/Y195F/W256C) and IDI. **a** The optimum pH was 7.5 measured at 25 °C. The mutations did not affect the optimal pH for IDI activity, but affect IDI activity for the optimal pH. **b** The general trends suggested that IDI (L141H/Y195F/W256C) had better pH stability propertied compared to wild-type IDI

As shown in Fig. 3a, IDI (L141H/Y195F/W256C) had higher activity than WT between pH 5.0 and pH 10.0. The activities of both enzymes increased when pH was < 7.5, and decreased when pH was > 7.5, suggesting that the optimum pH for both IDIs was 7.5 (Fig. 3a). The mutations did not affect the optimal pH for IDI activity, but affect IDI activity for the optimal pH. Figure 3b showed that in the pH range of 5.0–7.5, activity of IDI (L141H/Y195F/W256C) increased rapidly, and while the pH was > 7.5, activity decreased. The general trends suggested that IDI (L141H/Y195F/W256C) had better pH stability propertied compared to wild-type IDI.

**Fig. 4:**
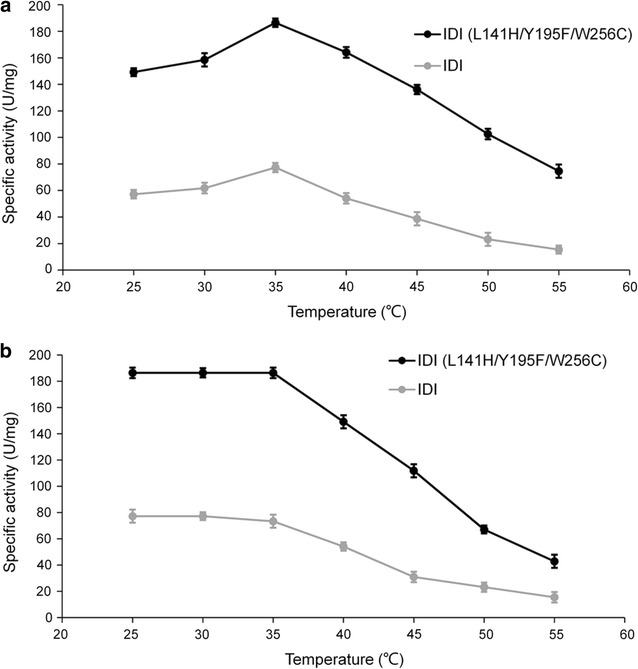
Effect of temperature on the activity (**a**) and stability (**b**) of IDI (L141H/Y195F/W256C) and IDI. **a** The optimal temperature was 35 °C, and that mutations did not affect the optimal temperature for IDI activity. **b** The thermal stabilities of both IDIs continuously decreased at temperature above 35 °C, while the thermal stability of wild-type IDI decreased more significantly than IDI (L141H/Y195F/W256C)

As shown in Fig. 4a, the enzymatic activities of wild-type and triple-mutant IDI increased from 25 to 35 °C, reaching a maximum at 35 °C. The activity of both IDIs were reduced at temperature above 35 °C, indicating that the optimal temperature for both IDI variants was 35 °C, and that mutations did not affect the optimal temperature for IDI activity. Interestingly, the activity of IDI (L141H/Y195F/W256C) was more than 100% compared to wild-type IDI at the optimal temperature. The thermal stabilities of both IDIs continuously decreased at temperature above 35 °C, while the thermal stability of wild-type IDI decreased more significantly than IDI (L141H/Y195F/W256C) (Fig. 4b). A possible explanation for this finding may be that a salt bridge was formed by the mutations, which enhanced the rigidity of the enzyme (Fig. 7b).

Mutants exhibited improved thermostability (Fig. 4b). Therefore, the thermostability of IDI (L141H/Y195F/W256C) was further analyzed by measuring the half-lives (T_1/2_) at 30–50 °C. Table 2 listed the T_1/2_ values of wild-type IDI and IDI (L141H/Y195F/W256C) at different temperatures. T_1/2_ at 25–50 °C were 2.6–6 times longer than the WT. Enhancement of the thermostability of IDI is particularly interesting, as it will extend the shelf life of IDI in the fermentation process.

**Table 2:** The half-lives (T_1/2_) of IDI and IDI (L141H/Y195F/W256C) at different temperatures

Temperature (°C)	IDI	IDI (L141H/Y195F/W256C)	Fold improvement
30	32.7 h (48.9%)	85.0 h (50.1%)	2.6
35	15.3 h (51.0%)	45.6 h (50.7%)	3.0
40	4.0 h (50.3%)	32.9 h (49.8%)	4.7
45	61 min (50.8%)	14.1 h (51.0%)	13.8
50	10 min (49.7%)	2.67 h (49.5%)	16.0

### Lycopene production of mutant and wild-type IDI in batch fermentation assays

The performance of IDI (L141H/Y195F/W256C) was ultimately evaluated in terms of the final lycopene yield and production through fed fermentation. For these experiments, the recombinant strain containing wild-type IDI was named CHL-1, and the recombinant strain containing IDI (L141H/Y195F/W256C) was named CHL-2. Fed-batch fermentations were carried out to test the suitability and stability of IDI (L141H/Y195F/W256C) for the improved production of lycopene.

**Fig. 5:**
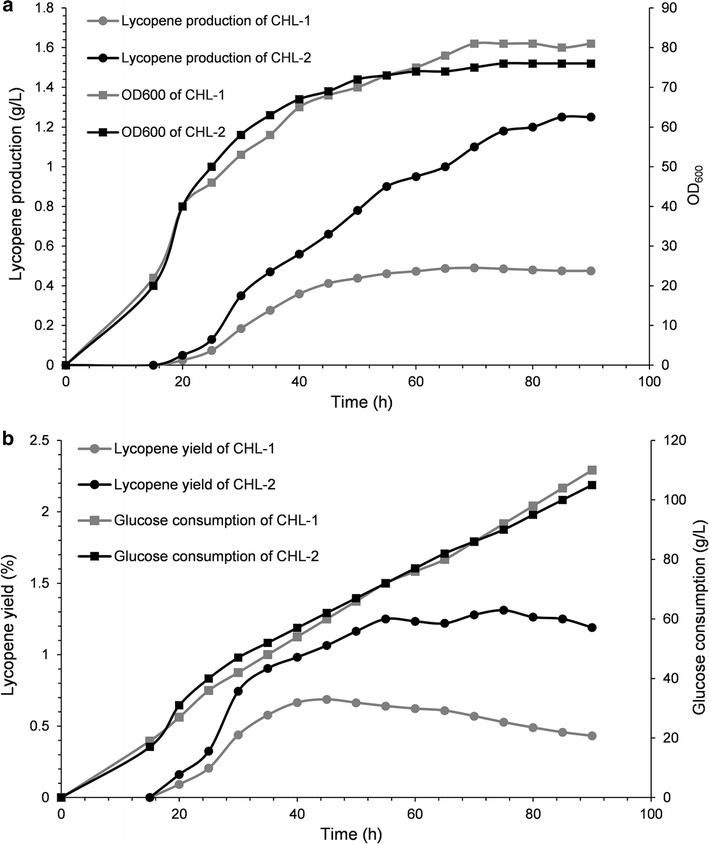
Cell growth rates, lycopene productions and lycopene yields, substrate consumption rates during aerobic fed-batch fermentation. **a** The cell growth rates of CHL-1 and CHL-2 were similar, and lycopene production of CHL-2 were approximately 2.8-fold of CHL-1. **b** The substrate rates of CHL-1 and CHL-2 were similar, while final lycopene yield of CHL-2 was approximately 3.1-fold of CHL-1

As shown in Fig. 5a, during the fed-batch stage, the biomass showed a steady increase, while the lycopene accumulation was significantly increased. After 90 h of fermentation, the strain CHL-2 had 1.8-fold-improved lycopene production above the original strain CHL-1 (Fig. 5a), while the lycopene yield of CHL-2 was 2.1-fold higher than that of CHL-1 (Fig. 5b). Although the fermentation process was stopped after 90 h, the lycopene accumulation in CHL-1 lasted for 70 h, while lycopene accumulation in CHL-2 was continuous throughout the whole fermentation process (Fig. 5a). The lycopene production and lycopene accumulation time suggested that the engineered triple-mutant IDI efficiently improved lycopene production.

### Structural simulation and molecular docking of IDI

**Fig. 6:**
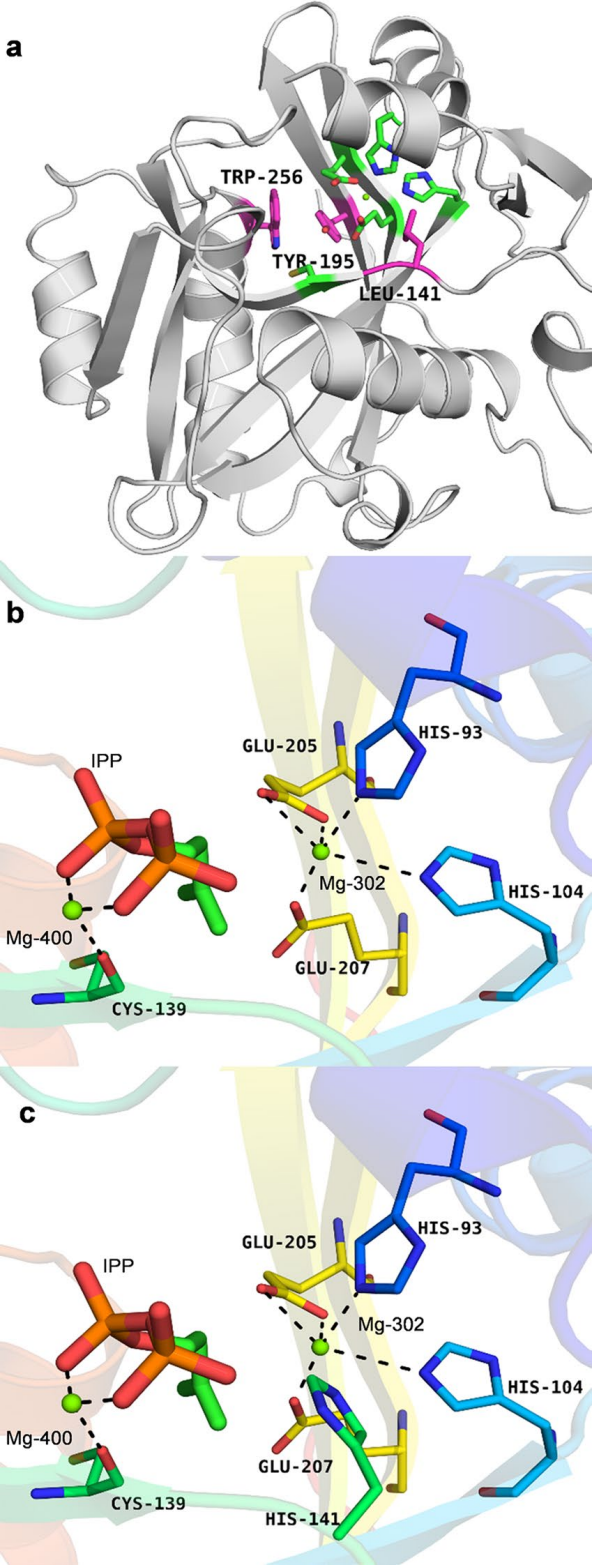
Structural models of IDI (**a**) and the mutation location: (**b**) L141; (**c**) L141H. **a** The location sites of the beneficial mutations were marked and the active site catalytic triad residues were marked in green. **b** Catalytic residues of activity pocket (His-93, His-104, Cys-139, Glu-205, Glu-207, Mg-302 and Mg-400) were marked respectively. **c** L141H substitution showed His-141 participated in the construction of the active pocket, which enlarged the IDI catalytic domain

**Fig. 7:**
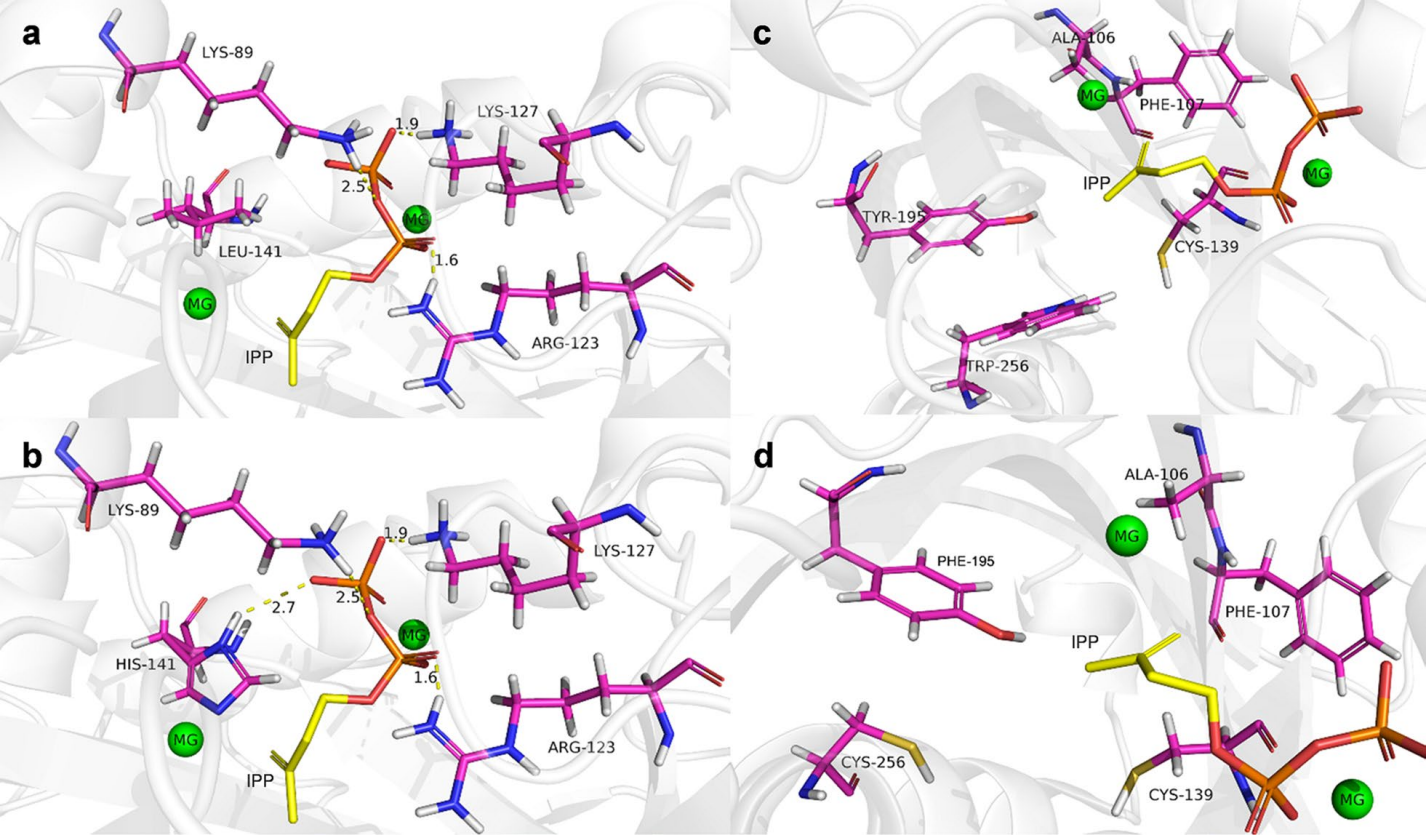
Structural models of mutation locations: **a** L141; **b** L141H; **c** Y195, W256; **d** Y195F, W256C. **a**, **b** L141H substitution showed His-141 formed a hydrogen bond with β-phosphates of IPP, strengthening binding of IPP. **c**, **d** Phe-195 and Cys-256 substitution was located near the hydrophobic group of IPP and improved the stability of binding of IPP

In order to provide insight into the molecular basis for increased enzymatic activity of IDI (L141H/Y195F/W256C), in silico structural modelling and molecular docking were performed by using the 3D structure of IPP isomerase from *E. coli* (PDB ID: 2ICJ), as a model template of IDI. The results of in silico structural modelling was shown in Fig. 6a. His-93, His-104, Cys-139, Glu-205, Glu-207, and Mg-302, Mg-400 formed an active pocket of IDI, identified to be the active sites (Fig. 6b). Cys-139 and Glu-207 were related to the protonation and deprotonation of substrate IPP, which were the most important sites of IDI [19]. In addition, the residual groups of Lys-89, Arg-123 and Lys127 were hydrogen-bonded to both α- and β-phosphates of IPP (Fig. 7a). Hydrophobic interaction of Ala-106, Phe-107 and Cys-139 with IPP could not only increase the binding capacity of enzyme and substrate, but also promote the correct refolding of protein (Fig. 7c). The residues at positions 141, 195 and 256, which are near the catalytic center of IDI in its 3D structure, would directly interact with the active center.

His-141 was located near the active pocket inside the tertiary structure of IDI (Fig. 6c). It was an alkaline amino acid, therefore it could bind the β-phosphate of IPP (negatively charged) (Fig. 7b). Binding of IPP was strengthened through the formation of additional hydrogen bond. Because of the hydrogen bond of His-141 and IPP, His-141 became one of the active sites (Fig. 6c) [20]. His-141 was near Mg-302 of the active pocket, and Mg-302 and His-141 had like electric charges. Thus Mg-302 repelled His-141, and it could be inferred that the hatchway for substrate molecules to enter the enzyme active center became larger and the hindrance reduced accordingly, thus enhancing the enzyme activity. The molecular docking results revealed that the binding energy between the substrate and the mutant enzyme was increased to 8.87 kcal/mol from the original 6.87 kcal/mol [21]. The increase in the binding energy could likely explain the improved substrate affinity.

Phe-195 and Cys-256 were nonpolar amino acids and located near the hydrophobic group of IPP (Fig. 7d). Due to hydrophobicity of Phe-195 and Cys-256, Ala-106, Phe-107 and Cys-139 could fixed substrate better [22]. The amino acid substitutions at the position of 195 and 256 enlarged the hydrophobic scope, and the active pocket indirectly therefore enhancing enzymatic activity to some extent.

## Discussion

The beneficial substitutions at positions 141, 195 and 256 were found by error-prone PCR (Fig. 2a) and the optimal amino acid substitutions at these positions were further analyzed to investigate their interactions and the molecular mechanisms behind this improved lycopene production (Fig. 2b). As shown in Fig. 2c, IDI worked better with any combination of single, double or triple mutations at these residues. This suggested that L141H, Y195F and W256C were distinct mutations that contribute considerably to the enhanced activity of mutant IDI.

Enzymatic properties showed that the three optimal substitutions, L141H, Y195F and W256C, were beneficial for the improved activity and thermostability of IDI. 2.53-fold improvement in catalytic activity suggested that three mutations enhanced performance of mutant IDI in the biocatalysis of substrates. Although the mutations did not affect the optimal pH and temperature for IDI, but affect IDI activity for the optimal pH and temperature significantly. This suggested that enhanced enzymatic activity was primarily a result of two main factors: (1) improved affinity between the substrate and enzyme caused by mutations (L141H, Y195F and W256C) and (2) increased enzyme stability due to mutations within the hydrogen bond and hydrophobic interaction [23]. In addition, the T_1/2_ values at 35 °C of IDI and IDI (L141H/Y195F/W256C) expressed in *E. coli* were 15.3 and 45.6 h, respectively, indicating that the hydrogen bond and hydrophobic interaction of L141H, Y195F, and W256C could improve its thermostability.

The fermentation results revealed enhanced performance of mutant IDI with regards to the production and final yield of target product. This was the ultimate goal of directed evolution of IDI. As shown in Fig. 5a, the strain CHL-2 had improved lycopene production compared to the original strain CHL-1, indicating that triple-mutant (L141H/Y195F/W256C) could translate improved enzymatic activity into the improvement of lycopene production successfully in an expanded system. For industrial application, this was a considerable advantage. Under these conditions, finally the lycopene yield of CHL-2 was higher than that of CHL-1 (Fig. 5b) [24]. This suggested that more glucose would be used to synthesize lycopene, another very important consideration for industrial application. This promised more substrate would be translated into target product rather than by-product, thus minimizing waste.

A 3D homology model of IDI was created to improve our understanding of the mutagenesis effect on IDI activity (Fig. 6a). According to the model, all mutations, L141H, Y195F and W256C are located near the activity pocket and may have interactions with IPP. Generally, three mutations have a cumulative effect for IDI (Fig. 2c). In this work, the respective change at position 141 could improve the structural stability through hydrogen bond (Fig. 7a, b), and increasing binding energy would be used to reduce the activation energy, to increase the substrate affinity, and the *K*_m_ value of IDI (L141H/Y195F/W256C) decreased (Table 1). The residues 195 and 256 with their surroundings (Ala-106, Phe-107 and Cys-139) (Fig. 7c) determine the conformation of IDI. The hydrophobic interactions of Phe195 and Cys256 with their surroundings had a better effect on the local structural stability (Fig. 7d) in comparison with those of Tyr195 and Trp256 (Fig. 7c). Therefore, Y195F/W256C may improve the thermostability of IDI.

## Conclusion

In conclusion, we have successfully engineered IDI to improve its catalytic activity and lycopene production via a random mutagenesis approach. In this study, we used error-prone PCR to obtain a mutant IDI library and developed a lycopene-dependent color development reaction to screen for activity-enhancing mutations. The best mutants produced by this approach maintained catalytic activity while also displaying improved thermo- and pH stability and longer half-life. These properties could make it a potent and attractive biocatalyst for the production of biochemicals. Fermentation experiments revealed that the lycopene production of triple-mutant IDI was 1.8-fold higher than wild-type IDI; however, due to the inherent deficiency of error-prone PCR, it was difficult to substantially increase IDI activity. The IDI performance in lycopene production could be further optimized by using additional or alternative protein engineering techniques, such as DNA shuffling [25], rational design [26], B-FIT [27], or ISM [28] approaches. The X-ray crystal structures of these engineered enzymes will also be further investigated to further elucidate the molecular mechanisms that govern catalytic activity.

## Methods

### Chemicals and materials

Restriction enzymes, T4 DNA ligase, Taq DNA polymerase, and PCR reagents were purchased from Takara Biomedical Technology (Beijing) Co., Ltd., and primers were synthesized by Jin Weizhi Biological Technology Co., Ltd. Gel Extraction Kit, PCR product DNA Purification Kit and Plasmid Mini Kit were from Omega Bio-Tek. All reagents and chemicals were of analytical grade and, unless otherwise stated, obtained from commercial sources.

### Bacterial strains and plasmids

Details of the plasmids and bacterial strains used in this study were shown in Additional file 1: Table S1. The *E. coli* strains BL21(DE3) and DH5α (Invitrogen) were used for plasmid preparation, protein overexpression/lycopene fermentation, respectively. Gene cloning was performed using *S. cerevisiae* (Invitrogen). Recombinant plasmid pCLpTrcUpper [29] was built and stored by our laboratory. Recombinant plasmid pAC-LYC [30] was provided by University of Maryland. Vector pETDeu-1 was purchased from Invitrogen.

### Plasmid construction

The four key enzymes that represent the bottom portion of the MVA pathway were combined for the lycopene color development reaction screening. Through the bottom pathway, the primary phosphorylation by mevalonate kinase (MK, EC2.7.1.366) and secondary phosphorylation by phospho-mevalonate kinase (PMK, EC2.7.4.2) converts MVA to diphosphomevalonate (DPMVA). Isomerization by isopentenyl diphosphate isomerase (IDI, EC5.3.3.2) and decarboxylation by diphospho-mevalonate decarboxylase (MVD, EC4.1.1.33) produces dimethylallyl pyrophosphate (DMAPP).

Four *S. cerevisiae* genes (PMK, MVD, MK, and IDI; ATCC201508D) were cloned into the pETDeut-1 vector. The pET-CHL1 plasmid was constructed by inserting PMK (*ERG8*) into the *Bgl* II and *Aat* II sites of pETDuet-1 with ERG8_F and ERG8_R primers. The plasmid pET-CHL2 was created by inserting MVD (*ERG19*) into the *Aat* II and *Xho* I sites of pET-CHL1 with primers ERG19_F and ERG19_R. The pET-CHL3 plasmid was established by inserting MK (*ERG12*) into the *Sac* I and *Not* I sites of the pET-CHL2 vector with primers ERG12_F and ERG12_R. The pET-CHL plasmid was built by the PCR amplification IDI (*IDI*) using primers IDI_F and IDI_R and cloning it into the *Sac* I and *Sal* I sites of the plasmid pET-CHL3.

### Construction of the mutation library

Error-prone PCR was used to introduce random mutations into *IDI* within the pET-CHL vector. The error-prone PCR reaction was as follows: 10× error-prone PCR buffer; 0.5 mmol/L dATP and dGTP; 2.5 mmol/L dCTP and dTTP; 40 pmol of each primer (IDI_F and IDI_R); 3 mmol/L MgCl_2_; 0.2 mmol/L MnCl_2_; 2.5 U Taq DNA polymerase, and water. The hot-start PCR amplification program annealed primers at 62 °C for 2 min, and product extension was performed at 72 °C for 2 min; this cycle was repeated 35 times.

After error-prone PCR amplification, PCR products and the pET-CHL vector were purified by *Sac* I and *Sal* I digestion, ligated, and transformed into competent DH5α *E. coli*, and positive colonies were selected and amplified to purify mutant plasmids.

### Screening of mutants with high lycopene production

The pET-CHL and pAC-LYC plasmids were transformed in *E. coli* BL21(DE3), and the transformation reaction was spread onto LB-agar plates, which were cultured for 15 h at 37 °C to generate the mutant screening library. Colonies were considered candidate strains and were cultured in 96-well plates in 300 μL of LB medium supplemented with MVA (3 g/L), ampicillin (100 μg/mL), and chloramphenicol (50 μg/mL) before incubation for 8 h at 37 °C. Protein expression was then induced with 0.05 mmol/L Isopropyl β-d-1-thiogalactopyranoside (IPTG) for 48 h, and then the cells were pelleted by centrifugation at 3000×*g* for 5 min. Next, the pellet was resuspended in 200 μL of acetone, and the mixture was incubated for 15 min at 55 °C, and then centrifuged at 10,000×*g* for 3 min. Finally, 200 μL of the supernatant was measured at OD_475_ on a spectrophotometer to indirectly determine the relative lycopene content [31]. Supernatant isolated from the control strain did not absorb light at 475 nm under the same conditions.

### Site-specific saturation mutagenesis

To construct IDI libraries containing all possible amino acid substitutions at residues L141, Y195, and W256 of IDI, the target codon was replaced with an NNK degenerate codon (K represents G or T and N represents A, T, G, or C) using the QuikChange II site-directed mutagenesis kit. The IDI-L141, IDI-Y195, and IDI-W256 libraries were produced with the L141-F/R, Y195-F/R, and W256-F/R primers, respectively (Additional file 1: Table S1). The constructs from all three libraries were then transformed into competent BL21(DE3). The original pET-CHL plasmid containing wild-type *IDI* was used as the template.

### Site-directed mutagenesis

The double mutations L141H/Y195F, L141H/W256C, Y195F/W256C and triple mutation L141H/Y195F/W256C were created by using the QuikChange II site-directed mutagenesis kit. The Y195F substitution was introduced into pET-CHL/IDI-L141H using Y195F-F and Y195F-R primers (see Additional file 1: Table S1). *E. coli* BL21(DE3) competent cells were transformed with the pET-CHL/IDI-L141H/Y195F plasmid using electroporation.

### IDI activity assay

The quantity of isoprene was measured by coupling the acidification and heating of DMAPP with the reactions catalyzed from IPP to DMAPP by IDI. This coupled reaction was monitored subsequently by gas chromatography (GC). For this, 2-mL sealed vials were used and added with 88 µL of protein extract, 2 µL of 1 M MgCl_2_, and 10 µL of 2.5 mM IPP in an assay buffer (50 mM MOPS, 20 mM MgCl_2_, 5% glycerol (v/v), pH 7.5). The vials were incubated at 30 °C for 90 min. The reaction mixtures were acidified by adding 10 µL of 85% H_3_PO_4_ and heated to 70 °C for 90 min. The resulting isoprene released from DMAPP was measured by GC [32].

### Determination of kinetic parameters and effect of temperature and pH on enzyme activity

Purified IDI was used to investigate the *V*_max_, *K*_m_, *K*_cat_, pH and thermal stability of the enzyme. The *K*_m_ value was determined by double-reciprocal plot method where the concentration range of the substrate was 1–1000 mmol/L [33]. Phosphate buffer (0.05 mol/L, pH 5.0–8.0), 0.05 mol/L Tris–HCl buffer (pH 8.0–9.0) and 0.05 mol/L carbonate buffer (pH 9.0–10.0) were prepared to determine the optimum pH required for IDI activity [34]. When determining the stability of IDI with altered pH conditions, 0.25 U enzyme solution was respectively added to the above buffer solutions with different pH values, and then the solutions were stored at 25 °C for 1 h to determine activity of the enzyme under standard conditions. When determining thermal stability, 0.25 U enzyme solution underwent heat preservation at 25–55 °C for 1 h to determine the IDI activity under standard conditions. In addition, the T_1/2_ of IDI and mutant IDI at 30–50 °C were measured. The systems containing 0.1 mg/mL purified IDI (0.05 mol/L phosphate buffer, pH 7.5) were utilized by incubating for different time intervals at different temperatures.

### Lycopene production by wild-type IDI and its variant in batch fermentation

A Biostat B plus MO5L fermenter (Sartorius Stedim Biotech GmbH, Göttingen, Germany) was used to perform fed-batch fermentation reactions using 2 L of fresh M9 medium (20 g glucose, 9.8 g KH_2_PO_4_, 2.1 g citric acid, 0.3 g ammonium ferric citrate, 5 g beef extract, 25 mg MgSO_4_·7H_2_O per 1 L water, pH 7.0) at 37 °C. After the initial carbon source (20 g/L glucose) was almost entirely consumed, a 3 M glucose solution was added to begin the fed batch mode. A 25% ammonia solution was used to maintain a constant neutral pH. Given that oxygen is an important factor for isoprenoid synthesis, fermentation was performed under strict aerobic conditions, with the dissolved oxygen concentration maintained at 20% saturation. When the OD_600_ reached 20, 0.05 mM IPTG was added to induce recombinant protein expression. Fresh IPTG and antibiotics were added every 24 h, and lycopene production and cell growth were monitored every 5 h throughout the 72 h fermentation process [35].

### Simulation of 3D structure and molecular docking of substrate of mutant IDI

The NCBI protein database (http://www.ncbi.nlm.nih.gov/protein/) was utilized to search the amino acid sequence of IDI from *S. cerevisiae*. The BLAST server (http://blast.ncbi.nlm.nih.gov) was utilized to search a template for the chain. We applied *E. coli* IDI (PDB ID: 2ICJ) as the template [36], and the homology of amino acid sequence was determined by sequence alignment. Homology modeling of IDI was carried out using SWISS-MODEL.

An in silico molecular docking analysis was executed to investigate potential binding modes between IDI and IPP using Autodock vina 1.1.2. The tertiary and quaternary structure of IDI was built by SWISS-MODEL. The 2D structure of IPP was drawn by ChemBioDraw Ultra 14.0 and converted to 3D structure using ChemBio3D Ultra 14.0 software [37, 38]. Docking input files were created with the AutoDockTools 1.5.6 package [39]. The search grid for IPP was determined as center_x: 42.508, center_y: 33.926, and center_z: 6.015 with dimensions size_x: 15, size_y: 15, and size_z: 15. The value of exhaustiveness was set to 20. Default Vina docking parameters were used unless otherwise described [40]. The best-scoring model as determined by the Vina docking score was visually analyzed using PyMoL 1.7.6 software (http://www.pymol.org/).

## Additional file

**Additional file 1.** Methods, Tables and Figures.

## Abbreviations

DMAPP: dimethylallyl pyrophosphate
GC: gas chromatography
IDI: isopentenyl diphosphate isomerase
IPP: isopentenylpyrophosphate
IPTG: isopropyl β-d-1-thiogalactopyranoside
MEP: 2-methyl-d-erythritol 4-phosphoric acid
MK: mevalonate kinase
MVA: mevalonate
MVD: diphosphomevalonate decarboxylase
PMK: phosphomevalonate kinase
