# Correction to: Enhancement of the catalytic activity of Isopentenyl diphosphate isomerase (IDI) from *Saccharomyces cerevisiae* through random and site-directed mutagenesis

**DOI:** 10.1186/s12934-019-1268-9

**Published:** 2020-01-13

**Authors:** Hailin Chen, Meijie Li, Changqing Liu, Haibo Zhang, Mo Xian, Huizhou Liu

**Affiliations:** 1 CAS Key Laboratory of Bio-based Materials, Qingdao Institute of Bioenergy and Bioprocess Technology, Chinese Academy of Sciences, No. 189 Songling Road, Qingdao, 266101 People’s Republic of China; 2 Sino-Danish College, University of Chinese Academy of Sciences, No. 19(A) Yuquan Road, Beijing, 100049 People’s Republic of China

## Correction to: Microb Cell Fact (2018) 17:65 10.1186/s12934-018-0913-z

The authors of this article [1] wish to draw the readers’ attention to their closely related paper, published in RSC Advances [2] which should have been cited in this article. The authors regret that there is unattributed overlap in text describing the construction of the plasmid coding for the biosynthetic pathway because of the commonly used research strategies between this article [1] and similar work presented in RSC Advances, although this does not affect the main scientific conclusion in this study.

The authors confirm that new data has been reported in this RSC Advances article. The two papers reported different key enzymes in the lycopene synthetic pathway to improve lycopene production. The MCF article used a directed evolution method to improve enzyme activity, half-life and substrate affinity of Isopentenyl diphosphate isomerase (IDI). Meanwhile, the RSC Advances article used the same method to improve the activity of mevalonate kinase to remove certain inhibition of lycopene biosynthesis intermediates. The catalytic mechanisms of both enzymes are different.

It should also be noted that data points were selected from raw data for presentation in Figure 2. The authors limited to show 20 colonies from each library of random and saturation mutagenesis based on the lycopene production (OD_475_). 20 isolated strains with the highest lycopene production were selected. Some strains with the similar lycopene production of the control were artificially abandoned, while strains in which the lycopene synthesis function were completely inactivated were not shown.
