# Supplementary material for: Enhancement of the catalytic activity of Isopentenyl diphosphate isomerase (IDI) from Saccharomyces cerevisiae through random and site-directed mutagenesis
Source: Microb Cell Fact. 2018 Apr 30;17:65. doi: 10.1186/s12934-018-0913-z (PMC5925831; doi:10.1186/s12934-018-0913-z)
Supplement: Supplementary file 1 — Additional file 1. Methods, Tables and Figures. [file 12934_2018_913_MOESM1_ESM.docx]

**Additional Material**

**Enhancement of the catalytic activity of Isopentenyl diphosphate isomerase (IDI) from *Saccharomyces cerevisiae* through random and site-directed mutagenesis**

Hailin Chen^1, 2^, Meijie Li^1^, Changqing Liu^1^, Haibo Zhang^1^, Mo Xian^1^, Huizhou Liu^1^

1. CAS Key Laboratory of Bio-based Materials, Qingdao Institute of Bioenergy and Bioprocess Technology, Chinese Academy of Sciences, No.189 Songling Road, Qingdao 266101, People’s Republic of China

2. Sino-Danish College, University of Chinese Academy of Sciences, No.19(A) Yuquan Road, Beijing 100049, People’s Republic of China

**Methods**

**Protein expression and purification**

IDI was expressed and purified from *E. coli* BL21(DE3) cells harboring the plasmid pET–IDI. The bacterial strain with IDI mutations were used to inoculate LB medium which was maintained at 37°C until the optical density at 600 nm (OD_600_) reached between 0.6 and 0.8 units. 0.2 mM IPTG was used to induce recombinant protein expression at 20°C. Cells were pelleted after 20 h by centrifugation at 10,000*g* and resuspended in 50 mM phosphate buffer (pH 7.4) containing 4 mM β-mercaptoethanol. Sonication was used to lyse the cells (3 s pulses with 3 s intervals between cycles at 60% output) at 4°C for a total of 40 min. Lysed cells were then clarified by centrifugation at 18,000*g* for 10 min, with samples kept at 4°C. A 0.22 µm PALL filter was used to further clarify the supernatant then loaded onto a nickel affinity chromatography column, which was pre-washed with 10 ml water and equilibrated with 10 ml binding buffer. Recombinant protein labelled with a 6 His-tag was able to bind to the nickel ions within the column. Sequential washing with 10 ml washing buffer 1 removed unbound protein from the column, while sashing Buffer 2 was used to remove nonspecific or weakly interacting, contaminating proteins. 10 ml elution fractions were collected and the concentration of each fraction was determined with the BCA protein assay quantification kit as per the manufacturer’s instructions. Recombinant protein was stored at −20°C after being flash frozen in liquid nitrogen.

**Lycopene quantification assay**

Lycopene was extracted from cellular fractions of each bacterial culture immediately after total glucose exhaustion. After washing, the pellet was extracted using acetone (1 ml) at 55°C and under intermittent vortexing for 15 min. The lycopene content in the supernatant was quantified by determination of the absorbance at 475 nm and calculated according to a standard curve. Extractions were completed in the dark to prevent photo-bleaching and degradation.

**Table S1.** Strains and plasmids used in this study.

| **Strain/plasmid/primer** | **Descriptions** | **Reference** |
| --- | --- | --- |
| Strains |  |  |
| *E. coli*  BL21(DE3) | *E. coli* str. B F^–^ *ompT* *gal* *dcm* *lon* *hsdS_B_*(*r_B_*^–^*m_B_*^–^) λ(DE3[*lacI* *lacUV5*-*T7p07* *ind1* *sam7* *nin5*]) [*malB*^+^]_K-12_(λ^S^) | Invitrogen |
| *E. coli*  DH5α | F^–^ *endA1* *glnV44* *thi-1* *recA1* *relA1* *gyrA96* *deoR* *nupG* *purB20* φ80d*lacZ*ΔM15Δ(*lacZYA-argF*)U169, hsdR17(*r_K_*^–^*m_K_*^+^), λ^–^ | Invitrogen |
| CHL-1 | BL21(DE3)/pET-CHL/pAC-LYC/ pCLpTrcUpper | This work |
| CHL-2 | BL21(DE3)/pET-CHL-IDI (L141H/195F/W256C)/pAC-LYC/ pCLpTrcUpper | This work |
| plasmids |  |  |
| pETDeu-1 | Ampicillin resistant; T7 promoter; has encoded N-terminal His6 tag | Invitrogen |
| pET-CHL1 | pETDeu-1 derivative carryinggenes gene *ERG8, T7* promoter, Ap ^R^ | This work |
| pET-CHL2 | pETDeu-1 derivative carryinggenes gene *ERG8* and *ERG19* , T7 promoter, Ap ^R^ | This work |
| pET-CHL3 | pETDeu-1 derivative carryinggenes gene *ERG8*, *ERG19* and *ERG12, T7* promoter, Ap ^R^ | This work |
| pET-CHL | pETDeu-1 derivative carryinggenes gene *ERG8*, *ERG19, ERG12* and *IDI, T7* promoter, Ap ^R^ | This work |
| pET-IDI | pET28a(+) derivative carryinggenes gene *IDI*, T7 promoter, Kan^R^ | This work |
| pCLpTrcUpper | pETDeu-1 derivative carryinggenes gene *mvaE* and *mvaS, T7* promoter, Spc ^R^ | [29] |
| pAC-LYC | pACYCDuet-1 derivative carryinggenes gene *crtE, crtI, crtB, T7* promoter, Cm ^R^ | [30] |
| Primers |  |  |
| ERG12_F | 5′-ACGCGTCGACTCATTACCGTTCTTAACTTC-3′ | |
| ERG12_R | 5′-ATTTGCGGCCGCTTATGAAGTCCATGGTAAAT-3′ | |
| ERG8_F | 5′-GGAAGATCTCTCAGAGTTGAGAGCCTTCAG-3′ | |
| ERG8_R | 5′-GGGCCGACGTCTTATTTATCAAGATAAGTTT-3′ | |
| ERG19_F | 5′-TCGCGACGTCACCGTTTACACAGCATCCGT-3′ | |
| ERG19_R | 5′-CCGCTCGAGTTATTCCTTTGGTAGACCAG-3′ | |
| IDI_F | 5′-CGAGCTCGACTGCCGACAACAATAGTAT-3′ | |
| IDI_R | 5′-ACGCGTCGACTTATAGCATTCTATGAATTT-3′ | |
| L141-F | 5'-CACATGCTGCTCTCNNKCACTATGTATTGATG-3' | |
| L141-R | 5'-CATCAATACATAGTGKNNGAGAGCAGCATGTG-3' | |
| Y195-F | 5'-CTTTTTAAACAGAATCCATNNKATGGCACCAAGCAATGAACC-3' | |
| Y195-R | 5'-GGTTCATTGCTTGGTGCCATKNNATGGATTCTGTTTAAAAAG-3' | |
| W256-F | 5'-GTTACAAGTTTACGCCTTNNKTTAAGATTATTTGCGAG-3' | |
| W256-R | 5'-CTCGCAAATAATCTTAAKNNAAGGCGTAAACTTGTAAC-3' | |
| L141H-F | 5'-CTAACACATGCTGCTCTCTTCCACTATGTATTGATG-3' | |
| L141H-R | 5'-CATCAATACATAGTGGAAGAGAGCAGCATGTGTTAG-3' | |
| Y195F-F | 5'-CTTTTTAAACAGAATCCATTGCATGGCACCAAGCAATGAACC-3' | |
| Y195F-R | 5'-GGTTCATTGCTTGGTGCCATGCAATGGATTCTGTTTAAAAAG-3' | |
| W256C-F | 5'-GTTACAAGTTTACGCCTTTGTTTAAGATTATTTGCG-3' | |
| W256C-R | 5'-CGCAAATAATCTTAAACAAAGGCGTAAACTTGTAAC-3' | |

**Table S2** Sequencing results of mutants.

| **Cycles** | **Mutant nucleotide bases** | **Mutant amino acids** |
| --- | --- | --- |
| 1 | A584G | Y195C |
| 2 | A584G/G767T | Y195C/W256L |
| 3 | A584G/G767T/T422A | Y195C/W256L/L141H |


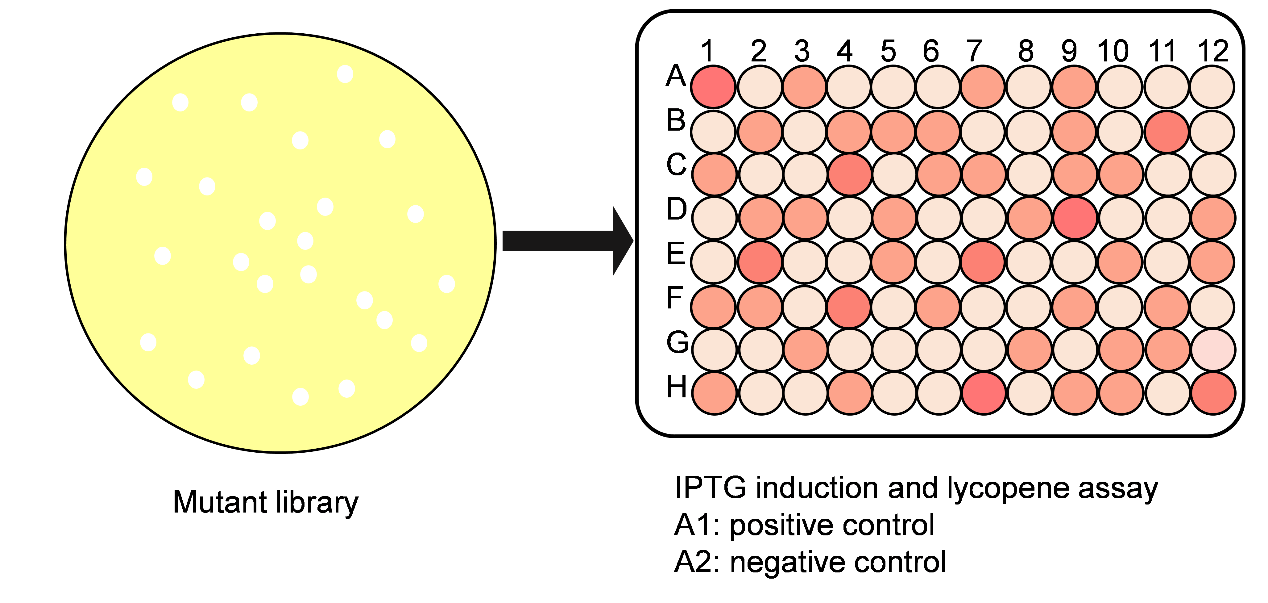


**Figure S1.** A two-step procedure for screening the IDI with improved lycopene production. Negative control: colonies with deactivated IDI; positive control: colonies with activated IDI.


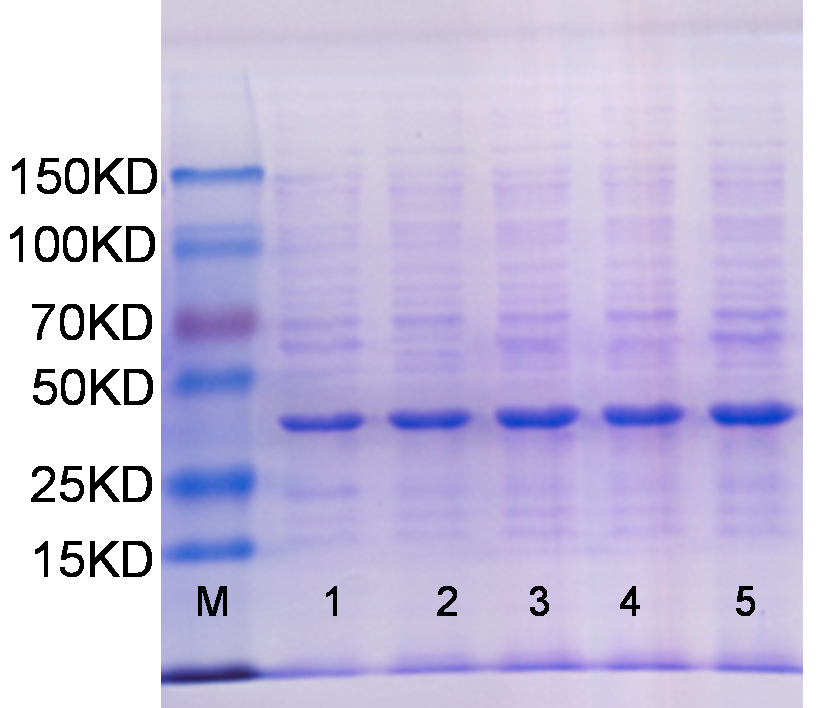


**Figure S2.** SDS-PAGE analysis of purified enzyme expressed in *E. coli* BL21(DE3). M, marker proteins; lane 1, wild type IDI; lane 2, IDI(L141H); lane3, IDI(Y195F); lane 4, IDI(W256C), lane 5, IDI(L141H/Y195F/W256C).
